# Cystoid Macular Edema in Non-Syndromic Retinitis Pigmentosa: Associations With Causative Genes in a Large Cohort

**DOI:** 10.1167/iovs.66.12.5

**Published:** 2025-09-03

**Authors:** Francesco Testa, Marianthi Karali, Rosa Boccia, Danila Pisani, Luciana Damiano, Antonio Nicolò, Emanuele Madonna, Luigi De Rosa, Raffaella Colucci, Antonella De Benedictis, Valentina Di Iorio, Paolo Melillo, Sandro Banfi, Francesca Simonelli

**Affiliations:** 1 Eye Clinic, Multidisciplinary Department of Medical, Surgical and Dental Sciences, University of Campania Luigi Vanvitelli, Naples, Italy; 2 Medical Genetics, Department of Precision Medicine, University of Campania Luigi Vanvitelli, Naples, Italy; 3 Department of Translational Medicine, University of Ferrara, Ferrara, Italy; 4 Telethon Institute of Genetics and Medicine, Pozzuoli, Italy

**Keywords:** cystoid macular edema (CME), non-syndromic retinitis pigmentosa, causative genes, large cohort

## Abstract

**Purpose:**

To investigate the prevalence of cystoid macular edema (CME) in relation to the disease-causing genes in a large cohort of genetically defined patients with non-syndromic retinitis pigmentosa (RP).

**Methods:**

Spectral-domain optical coherence tomography (SD-OCT) imaging has been retrospectively reviewed in order to assess the presence of CME over the disease course in a cohort of 580 patients with a clinical and genetic diagnosis of non-syndromic RP.

**Results:**

Over the course of the disease, 179 patients (30.9%) developed CME in at least one eye. Based on the patients’ genotypes, we found a statistically significant difference in CME prevalence according to the inheritance pattern (*P* < 0.001), with autosomal dominant forms being more frequently associated with CME (51.4%), followed by autosomal recessive forms (28.1%), but CME was rarely observed in X-linked RP (7.5%). By analyzing the most recurrent causative genes, we found the highest prevalence of CME in patients with autosomal dominant RP forms due to variants in *RHO* (58.2%), *PRPF8* (72.7%), and *PRPF3* (75.0%), whereas the lowest prevalence was observed in X-linked cases with mutations in *RP2* (3.4%) and *RPGR* (8.8%).

**Conclusions:**

This study revealed a strong association of CME with the underlying causative gene in non-syndromic RP in the largest genotyped cohort so far reported, adding new insights in the etiopathogenesis of CME in RP. Our findings emphasize the importance of SD-OCT morphological assessments of RP patients both to improve disease management and to better explore genotype–phenotype correlations.

Retinitis pigmentosa (RP) is a genetic disease that causes progressive degeneration of photoreceptors and retinal pigment epithelium (RPE), leading to gradual vision loss.^1^ It is a rare monogenic disorder with an estimated worldwide prevalence of 1:3500 to 1:4000, and it represents the most common subtype among inherited retinal diseases (IRDs) with about 2 million individuals currently affected.^1^^,^^2^ Commonly, RP occurs in an isolated/non-syndromic form confined to ocular manifestations, but it can also manifest as a syndromic form (in 20%–30% of cases) associated with a range of extraocular symptoms and multisystem involvement.^1^^,^^3^

The genetic etiology of RP is highly heterogeneous, with mutations in over 100 genes implicated in disease onset thus far (RetNet, http://retnet.org). The majority of RP-associated genes are linked to non-syndromic forms.^1^ Interestingly, pathogenic variants in some genes can be responsible for both isolated and syndromic RP forms, as exemplified by the involvement of biallelic *USH2A* genotypes in both isolated RP and Usher syndrome (OMIM *608400; http://omim.org/entry/608400).^4^^–^^6^ Autosomal recessive (AR) inheritance is the most prevalent, accounting for 50% to 60% of cases, with the *USH2A* gene being the most commonly affected.^1^^,^^4^^–^^6^ Autosomal dominant (AD) inheritance is observed in 30% to 40% of cases and is more frequently associated with variants in *RHO*.^1^^,^^4^^,^^7^ Finally, X-linked (XL) recessive forms account for 5% to 15% of cases, which primarily harbor disease-causing variants in *RPGR* (70%) and, to a lesser extent, in *RP2*.^1^^,^^4^^,^^8^

Advances in molecular diagnostics have enabled a genetic diagnosis in nearly 70% to 80% of RP patients, uncovering the extensive genetic and allelic heterogeneity that characterizes the disease.^9^^–^^11^ This heterogeneity provides valuable insights into the biochemical and cellular dysfunctions underlying the degeneration of rod photoreceptors in RP, which include defects in phototransduction, vitamin A metabolism, and intracellular trafficking, among other processes.^1^^,^^12^

Clinically, RP often begins with night blindness and constriction of the peripheral visual field, eventually leading to reduced central vision as the disease progresses and affects cone photoreceptors.^3^ A significant complication of RP is cystoid macular edema (CME), which affects approximately 10% to 58% of RP patients.^13^^–^^19^ CME is characterized by the presence of cystic spaces in the macula, leading to increased retinal thickness and exacerbating the already impaired vision in RP patients.^20^ The exact etiology of CME in RP remains unclear, but several hypotheses have been proposed.^16^^,^^20^^–^^24^ One theory suggests that the breakdown of the blood–retinal barrier, a structure crucial for maintaining retinal homeostasis, leads to the accumulation of fluid in the retina due to the release of toxic substances and inflammatory mediators such as vascular endothelial growth factor and interleukin-1.^21^^,^^22^ Another hypothesis focuses on the dysfunction of Müller cells, which are responsible for regulating potassium levels in the retina.^21^ Oxidative stress and inflammation may lead to the redistribution of potassium channels, resulting in Müller cell swelling and CME formation.^16^^,^^21^ Mechanical damage caused by vitreal traction has also been proposed as a contributing factor to CME, as it can exacerbate Müller cell dysfunction.^21^^,^^23^ Additionally, failure of the RPE pump mechanism may lead to fluid accumulation between the RPE and photoreceptors, contributing to CME.^21^ Finally, antiretinal antibodies have been detected in RP patients with CME, yet it is unclear whether they are a primary cause of CME pathogenesis or a secondary effect of retinal damage.^21^^,^^24^

Understanding the connection between RP causative genes and the development of CME could deepen our understanding of the pathogenetic mechanisms underlying this common complication. Previous efforts to identify such genetic associations have been limited to small patient cohorts,^15^^,^^25^^,^^26^ with the most extensive studies reporting findings from 103^17^ and 106^13^ RP patients with CME (from genotyped cohorts comprised of 578 and 145 patients, respectively). This study explored the correlation between disease-causing genes and CME in 179 patients identified from a large cohort of 580 genetically defined patients with non-syndromic RP that underwent spectral-domain optical coherence tomography (SD-OCT) imaging to assess retinal morphology. Our findings expand prior knowledge in the field and provide the basis for future studies that investigate the pathogenesis of CME with the aim of improving the management of RP patients, particularly in terms of CME treatment.

## Methods

### Ethical Statement

All procedures adhered to the tenets of the Declaration of Helsinki and were approved by the Ethics Board of the University of Campania Luigi Vanvitelli (for adults, protocol nos. 8189/2015, 09.04.2015; for pediatric subjects, protocol nos. 500/2017, 12.09.2017, 9130/2023, 24.03.2023). The cited protocols aimed to investigate genotype–phenotype correlations in IRDs. All patients provided informed consent for the data analysis. For minors, informed consent was obtained from the parents or legal guardians.

### Study Design

In this study, we included subjects who were recruited in the above-cited protocols between 2015 and 2024 at the Referral Center for Inherited Retinal Dystrophies (Eye Clinic of the University of Campania Luigi Vanvitelli, Naples, Italy) and satisfied the following inclusion criteria: a clinical diagnosis of non-syndromic RP and a conclusive genetic diagnosis. Data from clinical records were evaluated retrospectively to confirm the accuracy of the formulated diagnosis.^3^ For each included patient we analyzed the family history, anamnestic records, and SD-OCT assessments. SD-OCT was performed with a CIRRUS HD-OCT tomograph (Carl Zeiss Meditec, Dublin, CA, USA) using a 512 × 128 scan pattern, referred as the macular cube protocol (interscan distance of 46.8 µm) until 2016, and subsequently with the SPECTRALIS OCT Plus with the BluePeak module (Heidelberg Engineering, Heidelberg, Germany), obtaining a dense 20° × 15° volume scan centered on the fovea with 19 horizontal B-scans (interscan distance of 26.3 µm). To assess the presence of CME or other macular alterations, the SD-OCT scans of the entire cohort were reviewed independently by two retinal experts (RB, VDI) and adjudicated, in case of disagreement, by a third senior expert (FT). CME was defined as the presence of intraretinal hyporeflective spaces (>50 µm) with well-defined borders in two B-scan lines on SD-OCT macular volume, as done in a previous study.^27^

### Genetic Testing

The genetic diagnosis of the cohort was performed mainly by high-throughput genotyping methods using custom panels of IRD-associated genes or clinical exome or whole exome sequencing, as previously detailed.^10^ In some cases, the causative mutation was identified by Sanger confirmation of the disease-causing variants detected in other affected family members. The causative gene and inheritance pattern of the isolated RP, inferred by the patient's genotype and segregation data, were used to assess possible associations with CME.

### Statistical Analysis

The statistical analysis was performed using SPSS Statistics 25.0.0.0 (IBM, Chicago, IL, USA). Continuous variables (e.g., age) are expressed as mean ± standard deviation (SD) and were compared using the Student's unpaired *t*-test. Categorical variables (e.g., sex, inheritance pattern) are presented as counts and percentages. The frequency of CME in relationship with these variables was compared using the χ^2^ test. When appropriate, pairwise comparisons of column proportions were conducted using the *Z*-test for proportions, under the null hypothesis that the proportion of cases with CME in at least one eye is the same for each causative gene, and *P* values were adjusted for multiple comparisons using the Benjamini–Hochberg method. Moreover, logistic regression was fitted using generalized estimating equations models in order to investigate multivariate differences between patients with and without CME. Finally, the chi-square automatic interaction detection (CHAID) tree analysis was adopted to further explore the association between causative genes and CME. *P* < 0.05 was considered statistically significant.

## Results

### CME Prevalence and Genetic Composition of the Cohort

**Figure 1.**
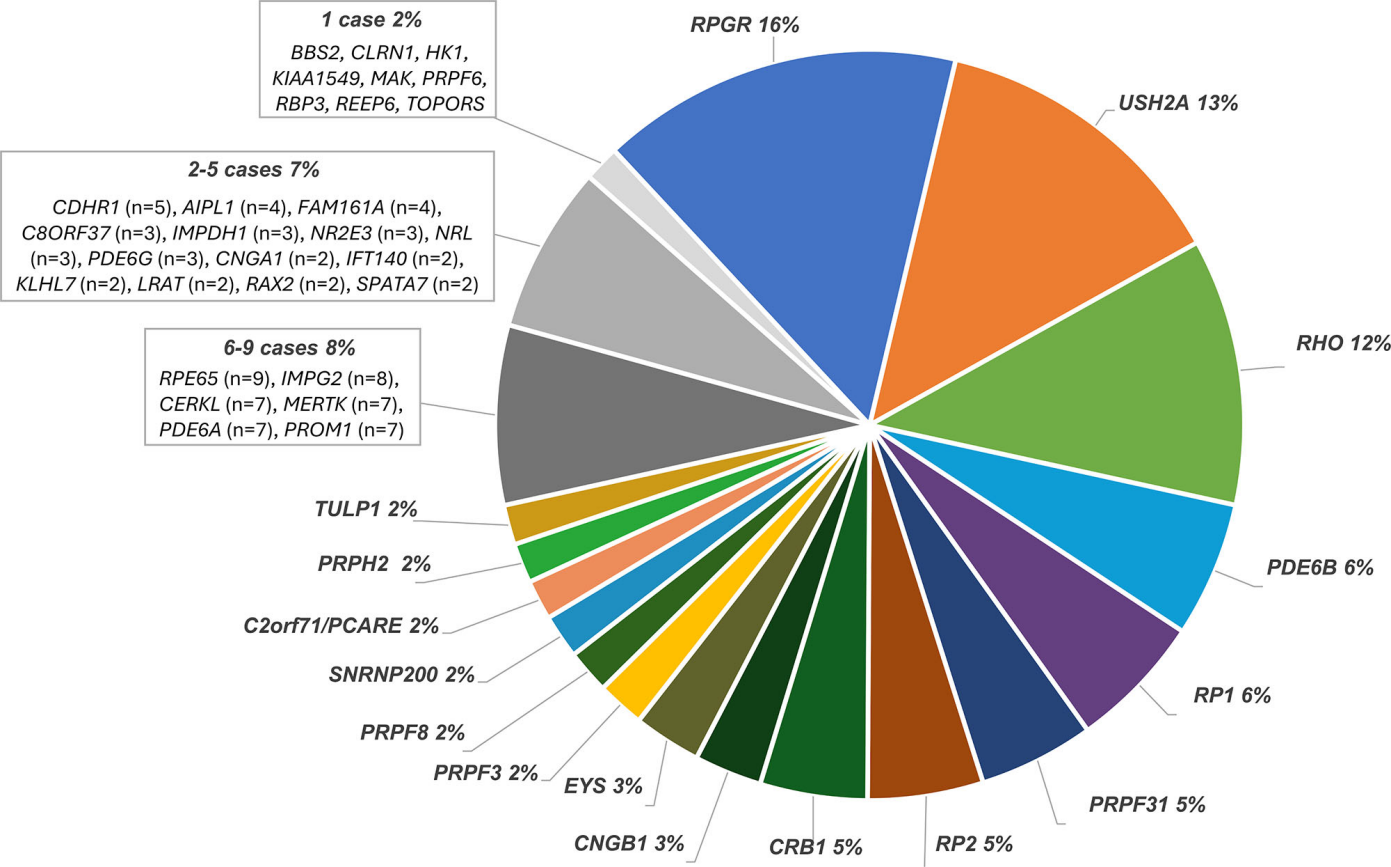
Causative gene frequency in the analyzed cohort. The pie chart shows the relative frequency of each of the 45 RP causative genes identified in the investigated cohort. Each causative gene, responsible for RP pathogenesis in at least 10 cases (*n* = 16), is plotted separately. Genes implicated in fewer than 10 cases (*n* = 29) are plotted as distinct groups (six to nine cases, two to five cases, and one case).

The study cohort included 580 genetically characterized patients with isolated RP with a mean age of 38.1 ± 17.6 years (range, 5–85 years). There was a slight prevalence of males (59.3%) in the RP cohort (344 males, 236 females), probably attributed to X-linked recessive RP cases. The genetic composition of the cohort confirmed the genetic heterogeneity of isolated RP, with 45 causative genes identified across the 580 patients (Fig. 1). In terms of the inheritance pattern, about 49.1% of the cases were AR forms (285 out of 580), 30.2% were AD (175 out of 580), and 20.7% were XL (120 out of 580). Among these genotyped patients, 179 individuals (30.9%) developed CME during their disease course (Supplementary Table S1). Of those, 124 patients manifested CME in both eyes (21.4%), and 55 patients developed monolateral CME (9.5%), for a total of 303 eyes with CME.

### CME Association With Age, Sex, and Inheritance Pattern

As summarized in Table 1, we then investigated the prevalence of CME in relation to age, sex, and inheritance pattern. In particular, no significant differences were observed in the age of patients presenting CME compared to those without CME (38.5 ± 16.7 years vs. 37.9 ± 18.0 years; *P* = 0.687). A statistically significant (*P* < 0.001) higher prevalence was observed in female patients (93 out of 236 subjects; 39.4%) compared to males (86 out of 344 subjects; 25.0%). Interestingly, we observed a statistically significant (*P* < 0.001) difference in the distribution of CME in patients stratified according to the inheritance pattern, with a higher prevalence of CME in AD forms (90 out of 175 subjects; 51.4%), followed by the AR ones (80 out of 285 subjects; 28.1%). On the other hand, the prevalence of CME in the X-linked forms was extremely low (nine out of 120 subjects; 7.5%). Therefore, we explored the relationship of sex and inheritance pattern by a multivariate logistic regression analysis. This model confirmed the different prevalence rates of CME among inheritance patterns (Wald χ^2^ test = 49.966; *P* < 0.001), whereas the differences observed between sexes did not reach statistical significance (Wald χ^2^ test = 0.523; *P* = 0.469). Indeed, these findings were confirmed by a stratified analysis by inheritance pattern, which showed that CME prevalence was similar between females and males with the same inheritance pattern (i.e., AR or AD), whereas for XL this comparison was not possible as the cohort did not include any XL RP-manifesting female carriers (Table 1).

**Table 1.** CME Prevalence (In at Least One Eye) in Relation to Age, Sex, and Inheritance Pattern

	Patients With CME (*n* = 179)	Patients Without CME (*n* = 401)	Test	Statistic	*P*
Age (y), mean ± SD	38.5 ± 16.7	37.9 ± 18.0	*t*-Test	−0.404	0.687
Sex, *n* (%)			χ^2^ test	13.616	<0.001
Male	86 (25)	258 (75)			
Female	93 (39.4)	143 (60.6)			
Inheritance pattern, *n* (%)			χ^2^ test	66.427	<0.001
AD	90 (51.4)	85 (48.6)			
AR	80 (28.1)	205 (71.9)			
XL	9 (7.5)	111 (92.5)			
Inheritance pattern and sex, *n* (%)					
AD, male	33 (43.4)	43 (56.6)	Stratified analysis	3.448	0.063
AD, female	57 (57.6)	42 (42.4)	by inheritance		
AR, male	44 (29.7)	104 (70.3)	pattern; χ^2^ test	0.420	0.517
AR, female	36 (26.3)	101 (73.7)			
XL, male	9 (7.5)	111 (92.5)		NA	NA

NA, not applicable.

### CME and Causative Gene Association

**Figure 2.**
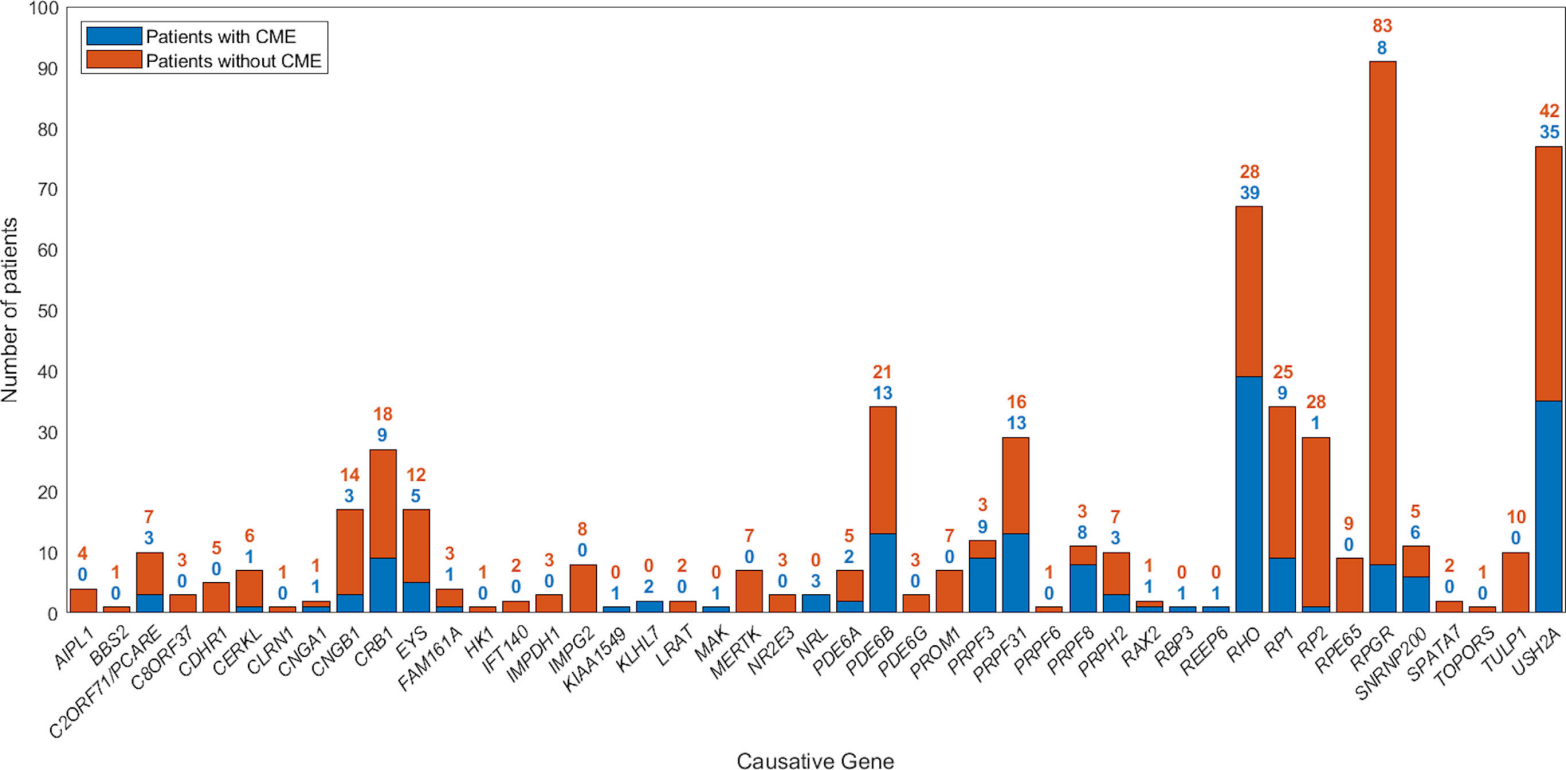
Frequency of CME across causative genes in the analyzed cohort. The histogram illustrates CME occurrence in non-syndromic RP patients with mutations in the most recurrent causative genes. The *height of the bar* indicates the total number of cases linked to each causative gene. The fraction of CME-presenting cases is highlighted in *blue*.

We explored the relationship of CME with the causative gene (Fig. 2). Interestingly, the statistical analysis showed differences in the distribution of CME depending on the RP-causing genes (Fisher exact test based on 10,000 sampled tables: 140.7; *P* < 0.001). In particular, we observed significantly higher prevalence of CME in patients with mutations in *PRPF3* (nine out of 12; 75.0%), *PRPF8* (eight out 11; 72.7%), *RHO* (39 out of 67; 58.2%), and *USH2A* (35 out of 77; 45.5%), whereas significantly lower prevalence was observed in *RP2* (1 out of 29; 3.4%) and *RPGR* (eight out of 91; 8.8%) associated X-linked cases (Table 2).

**Table 2.** CME Prevalence (In at Least One Eye) Across Genes

Gene	Cases, *n*	Cases With CME Presence, *n*	Cases With CME Absence, *n*	CME Presence	Adjusted P*
*AIPL1*	4	0	4	0.0%	^†^
*BBS2*	1	0	1	0.0%	^†^
*C2ORF71*/*PCARE*	10	3	7	30.0%	>0.05
*C8ORF37*	3	0	3	0.0%	^†^
*CDHR1*	5	0	5	0.0%	^†^
*CERKL*	7	1	6	14.3%	>0.05
*CLRN1*	1	0	1	0.0%	^†^
*CNGA1*	2	1	1	50.0%	>0.05
*CNGB1*	17	3	14	17.6%	>0.05
*CRB1*	27	9	18	33.3%	>0.05
*EYS*	17	5	12	29.4%	>0.05
*FAM161A*	4	1	3	25.0%	>0.05
*HK1*	1	0	1	0.0%	^†^
*IFT140*	2	0	2	0.0%	^†^
*IMPDH1*	3	0	3	0.0%	^†^
*IMPG2*	8	0	8	0.0%	^†^
*KIAA1549*	1	1	0	100.0%	^†^
*KLHL7*	2	2	0	100.0%	^†^
*LRAT*	2	0	2	0.0%	^†^
*MAK*	1	1	0	100.0%	^†^
*MERTK*	7	0	7	0.0%	^†^
*NR2E3*	3	0	3	0.0%	^†^
*NRL*	3	3	0	100.0%	^†^
*PDE6A*	7	2	5	28.6%	>0.05
*PDE6B*	34	13	21	38.2%	>0.05
*PDE6G*	3	0	3	0.0%	^†^
*PROM1*	7	0	7	0.0%	^†^
*PRPF3*	12	9	3	75.0%	**0.001**
*PRPF31*	29	13	16	44.8%	>0.05
*PRPF6*	1	0	1	0.0%	^†^
*PRPF8*	11	8	3	72.7%	**0.003**
*PRPH2*	10	3	7	30.0%	>0.05
*RAX2*	2	1	1	50.0%	>0.05
*RBP3*	1	1	0	100.0%	^†^
*REEP6*	1	1	0	100.0%	^†^
*RHO*	67	39	28	58.2%	**<0.001**
*RP1*	34	9	25	26.5%	>0.05
*RP2*	29	1	28	3.4%	**0.001**
*RPE65*	9	0	9	0.0%	^†^
*RPGR*	91	8	83	8.8%	**<0.001**
*SNRNP200*	11	6	5	54.5%	>0.05
*SPATA7*	2	0	2	0.0%	^†^
*TOPORS*	1	0	1	0.0%	^†^
*TULP1*	10	0	10	0.0%	^†^
*USH2A*	77	35	42	45.5%	**0.003**

* Each pair of columns was compared with *Z*-tests for proportion under the null hypothesis that the proportion of cases with CME in at least one eye is the same for each causative gene, and the relative *P* values were adjusted for multiple comparisons (*n* = 21) using the Benjamini–Hochberg method. Bold font denotes statistically significant associations.

† These categories are not included in the comparison because the frequency is equal to 0% or 100%.

**Figure 3.**
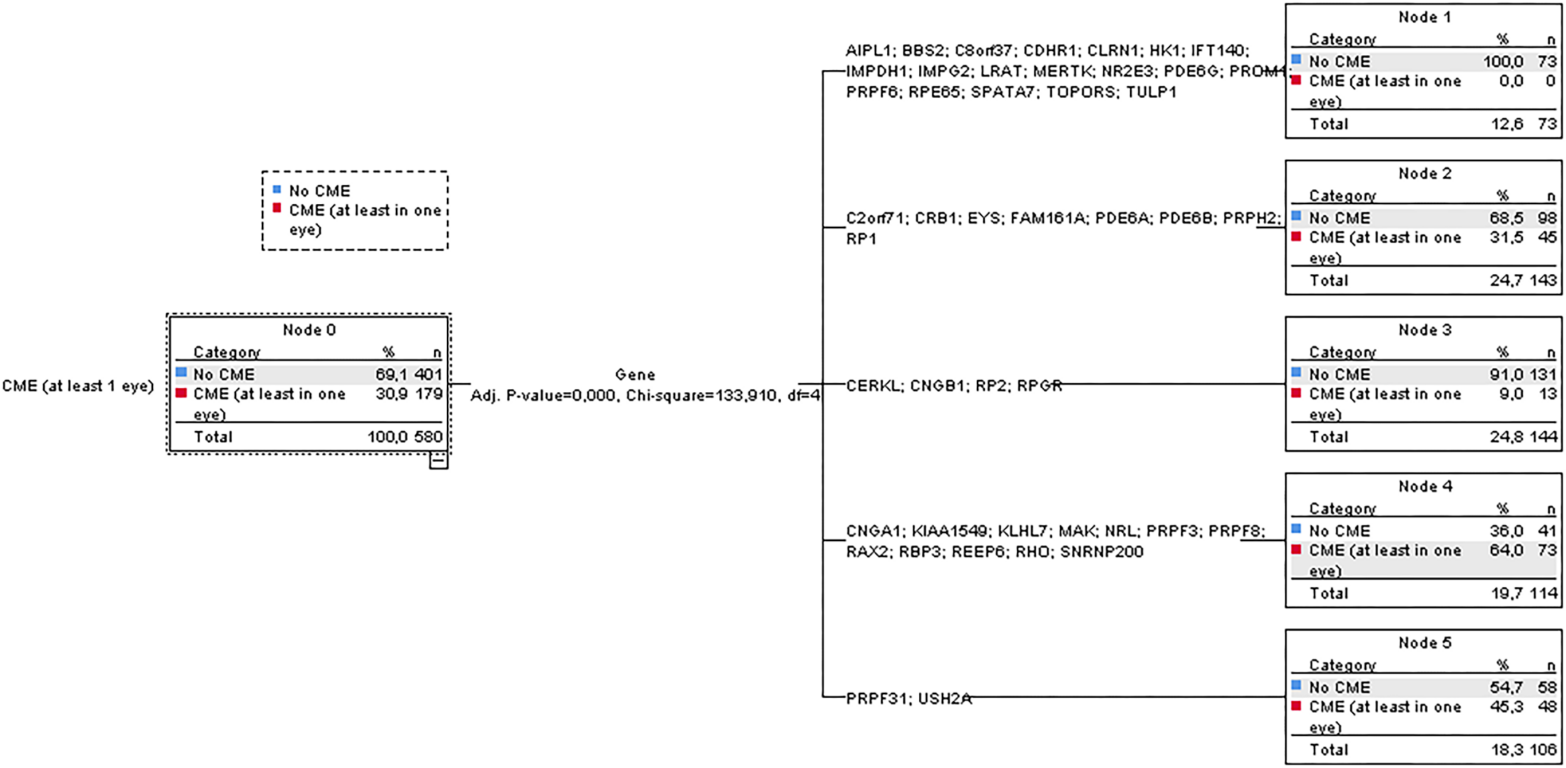
Associations between genotype and CME prevalence, using the CHAID tree model.

The CHAID tree model (Fig. 3) grouped the genes as follows: (1) genes with an extremely higher than average prevalence (about 50% or more) included *CNGA1* (50.0%), *RAX2* (50.0%), *SNRNP200* (54.5%), *RHO* (58.2%), *PRPF8* (72.7%), *PRPF3* (75.0%), *KIAA1549* (100%), *KLHL7* (100%), *MAK* (100%), *NRL* (100%), *RBP3* (100%), and *REEP6* (100%); (2) genes with a higher than average prevalence (40%–45%) included *PRPF31* (44.8%) and *USH2A* (45.5%); (3) genes with similar to average prevalence (25%–38%) included: *FAM161A* (25.0%), *RP1* (26.5%), *PDE6A* (28.6%), *EYS* (29.4%), *PRPH2* (30.0%), *C2ORF71/PCARE* (30.0%), *CRB1* (33.3%), and *PDE6B* (38.2%); (4) genes with a lower than average prevalence (lower than 18%) included *RP2* (3.4%), *RPGR* (8.8%), *CERKL* (14.3%), and *CNGB1* (17.6%); and (5) genes without CME cases included *AIPL1* (0%), *BBS2* (0%), *C8ORF37* (0%), *CDHR1* (0%), *CLRN1* (0%), *HK1* (0%), *IFT140* (0%), *IMPDH1* (0%), *IMPG2* (0%), *LRAT* (0%), *MERTK* (0%), *NR2E3* (0.0%), *PDE6G* (0%), *PROM1* (0%), *PRPF6* (0%), *RPE65* (0%), *SPATA7* (0%), *TOPORS* (0%), and *TULP1* (0%).

## Discussion

This retrospective longitudinal study investigated the prevalence of CME in 580 genetically characterized patients with non-syndromic RP and explored potential associations between specific genetic variants and CME occurrence. The CME prevalence in our cohort was 30.9%, in line with the majority of previous reports that have estimated CME frequency in RP cohorts to be between 20.4% and 58.6%.^13^^,^^14^^,^^17^^,^^26^^,^^28^^–^^30^ Only Arias et al.^13^ recently reported a higher prevalence of 73.1%. Interestingly, unlike the earlier reports on equally large RP cohorts that did not specifically consider the causative genes,^14^^,^^15^^,^^30^^,^^31^ this study highlights a strong genetic correlation with CME, revealing significant variability in prevalence depending on the mutated gene.

By considering the patients’ genotype, our analysis demonstrated a higher prevalence of CME in AD forms of RP, particularly those associated with mutations in *PRPF3*, *PRPF8*, and *RHO*. This CME association with dominant forms agrees with previous studies that inferred the mode of inheritance mainly from pedigree information.^14^^,^^15^^,^^17^^,^^32^

In our cohort, 75% of patients (nine out of 12) harboring pathogenic variants in *PRPF3* developed CME over the disease course. All of those were heterozygous for the p.Thr494Met missense change, suggesting a strong association of this variant with CME development. A previous report on a Swiss family segregating *PRPF3-*associated RP due to the same variant did not detect CME, but SD-OCT imaging was performed in only four of the 11 affected members.^33^ We observed an equally high CME prevalence (72.7%) among patients with mutations in *PRPF8* (p.Gln2181Pro, p.His2309Arg, p.His2313Pro, p.Tyr2326*). In relation to our findings, at least two earlier studies have documented the presence of CME in 40% of affected subjects (10 out of 25 cases) segregating pathogenic *PRPF8* variants.^34^^,^^35^ In that respect, we cannot exclude confounding effects due to the small number of patients carrying each variant or due to shared genetic modifiers in affected members of the same family that may influence their predisposition to CME development. In any case, the lack of SD-OCT morphological data in large cohorts of RP patients with mutations in the above-mentioned genes precluded a systematic comparison of our findings with the available literature.

More importantly, we identified a significant, positive association of CME with causative variants in *RHO* by analyzing a substantial sample size (*n* = 67 patients) (Table 2). Specifically, 58.2% of *RHO*-linked cases (39 out of 67 cases) developed CME, a proportion comparable to that reported by Nguyen et al.^36^ (50%; 16 out of 32 cases), whereas Arias et al.^13^ observed CME in a smaller fraction of *RHO* cases (35.7%; five out 14 cases). The CME prevalence varied across different *RHO* mutations (Table 3); however, reliable correlations could not be made due to the low sample size per variant. Furthermore, we evaluated the CME frequency on the basis of phenotype exhibited by the *RHO* patients as adopted in previous studies,^37^^–^^40^ and we observed similar CME frequency among class A and class B (20/32 [62.5%] vs. 19/35 [54.3%]; *P* = 0.463). The CME frequency rates in patients carrying the most recurrent *RHO* variants (namely, p.Arg135Trp and p.Pro347Leu) were 61.5% and 66.6%, respectively. The p.Pro347Leu mutation has already been associated with early and aggressive CME development in multiple studies.^41^^–^^43^ Our findings further support this association, with six out of nine patients exhibiting CME and the remaining three showing severe foveal atrophy on SD-OCT scans, a feature probably resulting from the earlier presence of CME. As for the p.Arg135Trp, although several studies described *RHO* patients with this hot-spot mutation,^36^^,^^38^^,^^40^^,^^44^^,^^45^ SD-OCT findings are scarce and fragmented to allow reliable comparisons.

**Table 3.** Variant-Specific CME Prevalence in *RHO*-Associated RP Patients

Allele			
Nucleotide Change^*^	Protein Change^†^	Phenotypic Class Associated With the Variant	Patients With CME/Total Number of Patients, *n* (%)
c.403C>T	p.(Arg135Trp)	A^38^^,^^39^	8/13 (61.5)
c.473C>A	p.(Ala158Asp)	B^‡^	8/13 (61.5)
c.1040C>T	p.(Pro347Leu)	A^39^	6/9 (66.6)
c.644C>T	p.(Pro215Leu)	A^‡^	4/5 (80)
c.541G>A	p.(Glu181Lys)	A^39^	1/3 (33.3)
c.499T>C	p.(Cys167Arg)	B^‡^	0/3 (0)
c.560G>T	p.(Cys187Phe)	B^‡^	2/2 (100)
c.509C>G	p.(Pro170Arg)	B^‡^	2/2 (100)
c.50C>G	p.(Thr17Arg)	B^‡^	1/2 (50)
c.364G>A	p.(Glu122Lys)	B^‡^	1/2 (50)
c.511C>T	p.(Pro171Ser)	B^‡^	0/2 (0)
c.538C>T	p.(Pro180Ser)	B^‡^	0/2 (0)
c.190C>T	p.(Gln64*)	B^39^	1/1 (100)
c.181G>A	p.(Val61Ile)	B^‡^	1/1 (100)
c.491C>T	p.(Ala164Val)	A^37^	1/1 (100)
c.568G>T	p.(Asp190Tyr)	B^39^	1/1 (100)
c.1045T>C	p.(*349Glnext*51)	B^‡^	1/1 (100)
c.1028G>A	p.(Ser343Asn)	B^‡^	1/1 (100)
c.44A>G	p.(Asn15Ser)	B^40^	0/1 (0)
c.545G>A	p.(Gly182Asp)	B^‡^	0/1 (0)
c.706C>A	p.(Gln236Lys)	A^‡^	0/1 (0)

* Variants are reported to the NM_000539.3 RefSeq transcript.

† Variants are reported to the P08100 sequence.

‡ Classified by this study.

Other RP-causative genes, such as *PRPF31*, *PDE6B*, and *USH2A*, showed CME prevalence rates of 44.8%, 38.2%, and 45.5%, respectively. Similar to our findings, Kim et al.^26^ reported a 50% CME prevalence in *PDE6B*-linked cases and a 33% prevalence in *USH2A* patients. The CME frequency in *USH2A*, *RHO*, and *PRPF* family of genes was consistent with the common association of these gene with inflammatory manifestations reported by the recent study by Sarici et al.^46^ Finally, the *RP1* gene showed a CME prevalence of 26.5% in our cohort (nine out of 34), whereas Arias et al.^13^ reported a significantly higher CME prevalence (83%; 10 out of 12). We found a similar discrepancy of CME frequency in *EYS* cases. In our study, CME frequency was 29.4% (five out of 17), close to that observed by Sarici et al.^46^ (15%; three out of 20), whereas Arias et al.^13^ reported 88% in the same sample size (*n* = 17), underscoring how differences in cohort composition (e.g., in terms of ethnicity or variant distribution) can impact the observed associations.

Interestingly, patients with mutations in the X-linked genes *RP2* and *RPGR*, despite representing a significant fraction of the cohort (*n* = 120), displayed virtual absence of CME (only nine). Previous studies have similarly reported a lack of CME in *RPGR* patients,^47^^,^^48^ as well as a low CME prevalence in XL RP forms.^14^^,^^32^^,^^48^

Taken together, the heterogeneity of the genetic causes associated with CME suggest that different pathophysiological mechanisms may underlie CME development in RP, warranting further investigation. It is intriguing that the CME development had the strongest association with mutations in genes encoding splicing factors (e.g., *PRPF3*, *PRPF31*, and *PRPF8* collectively showed 57.7% CME frequency; 30 out of 52 cases). Such variants are expected to cause spliceosome dysregulation, with downstream effects on the proper splicing of other genes and perturbed protein synthesis in the retina.

The current study presents the following limitations: Fluorescein angiography was not consistently performed in our RP patients, as it is not included in the diagnostic clinical pathway routinely adopted; therefore, there were no information about leakage. Given that only cystic changes larger than 50 µm were considered as evidence of CME and considering the inherent interscan variability in the available OCT scans, we cannot fully exclude that some cases of CME may have been underdiagnosed. Finally, syndromic forms of RP were not included.

In conclusion, this study contributes to efforts aimed at deciphering the potential role of genetic factors in the etiopathogenesis of CME in RP by describing findings in the largest genetically characterized cohort with non-syndromic RP so far reported. Our results emphasize the importance of SD-OCT morphological assessments in RP patients both to improve disease management and to better explore genotype–phenotype correlations. Although the exact mechanisms by which specific mutations contribute to CME remain unclear, our findings suggest that CME prevalence in RP is highly gene dependent. Further research is needed to elucidate the underlying mechanisms and eventually lead to new therapeutic targets for the treatment of CME.

## Supplementary Material

Supplement 1
