# Supplementary material for: Cystoid Macular Edema in Non-Syndromic Retinitis Pigmentosa: Associations With Causative Genes in a Large Cohort
Source: Invest Ophthalmol Vis Sci. 2025 Sep 3;66(12):5. doi: 10.1167/iovs.66.12.5 (PMC12410279; doi:10.1167/iovs.66.12.5)
Supplement: Supplement 1 [file iovs-66-12-5_s001.pdf]

Supplementary Table 1. Genotypes of non-syndromic RP patients presenting cystoid macular edema (CME) in at least one eye

Patient ID	Sex	CME presence	Causative Gene	Transcript (RefSeq)	Variant 1 (DNA change)	Variant 1 (Protein change)	Variant 2 (DNA change)	Variant 2 (Protein change)	Inheritance pattern
P1	Male	Monolateral	*C2ORF71/PCARE*	NM_001029883.3	c.2630G>A	p.(Trp877*)	c.2918C>G	p.(Ser973*)	AR
P2	Female	Bilateral	*C2ORF71/PCARE*	NM_001029883.3	c.2756_2768del	p.(Lys919Thrfs*2)	c.2756_2768del	p.(Lys919Thrfs*2)	AR
P3	Female	Bilateral	*C2ORF71/PCARE*	NM_001029883.3	c.3002G>A	p.(Trp1001*)	c.400_401del	p.(Ser134*)	AR
P4	Female	Monolateral	*CERKL*	NM_001030311.3	c.847C>T	p.(Arg283*)	c.847C>T	p.(Arg283*)	AR
P5	Male	Monolateral	*CNGA1*	NM_000087.5	c.668G>A	p.(Gly223Glu)	c.668G>A	p.(Gly223Glu)	AR
P6	Male	Bilateral	*CNGB1*	NM_001297.5	c.1535G>A	p.(Arg512Lys)	c.2296T>C	p.(Cys766Arg)	AR
P7	Male	Bilateral	*CNGB1*	NM_001297.5	c.290+1G>A	p.(?)	c.2903G>A	p.(Arg968Gln)	AR
P8	Male	Bilateral	*CNGB1*	NM_001297.5	c.838-2A>C	p.(?)	c.838-2A>C	p.(?)	AR
P9	Female	Bilateral	*CRB1*	NM_201253.3	c.1313G>A	p.(Cys438Tyr)	c.1313G>A	p.(Cys438Tyr)	AR
P10	Female	Monolateral	*CRB1*	NM_201253.3	c.1313G>A	p.(Cys438Tyr)	c.1313G>A	p.(Cys438Tyr)	AR
P11	Male	Bilateral	*CRB1*	NM_201253.3	c.1958T>C	p.(Ile653Thr)	c.1958T>C	p.(Ile653Thr)	AR
P12	Male	Bilateral	*CRB1*	NM_201253.3	c.1958T>C	p.(Ile653Thr)	c.1958T>C	p.(Ile653Thr)	AR
P13	Male	Bilateral	*CRB1*	NM_201253.3	c.2308G>A	p.(Gly770Ser)	c.2676+1_2676+2del	p.(?)	AR
P14	Female	Bilateral	*CRB1*	NM_201253.3	c.2308G>A	p.(Gly770Ser)	deletion exons 1-5	p.(?)	AR
P15	Male	Bilateral	*CRB1*	NM_201253.3	c.2843G>A	p.(Cys948Tyr)	c.1445_1453del	p.(Ile482_Tyr484del)	AR
P16	Male	Bilateral	*CRB1*	NM_201253.3	c.498_506del	p.(Ile167_Gly169del)	c.2234C>T	p.(Thr745Met)	AR
P17	Male	Bilateral	*CRB1*	NM_201253.3	c.683G>A	p.(Cys228Tyr)	c.3751C>T	p.(Gln1251*)	AR
P18	Female	Bilateral	*EYS*	NM_001142800.2	c.5916dup	p.(Leu1973Thrfs*11)	c.5959A>C	p.(Thr1987Pro)	AR
P19	Female	Monolateral	*EYS*	NM_001142800.2	c.6571+1G>A	p.(?)	c.32dup	p.(Met12Aspfs*14)	AR
P20	Female	Monolateral	*EYS*	NM_001142800.2	c.9071T>G	p.(Leu3024Trp)	c.9071T>G	p.(Leu3024Trp)	AR
P21	Female	Monolateral	*EYS*	NM_001142800.2	c.9299_9302del	p.(Thr3100Lysfs*26)	c.5928-2A>G	p.(?)	AR
P22	Male	Monolateral	*EYS*	NM_001142800.2	c.9428_9429dup	p.(Thr3144Leufs*42)	c.9428_9429dup	p.(Thr3144Leufs*42)	AR
P23	Male	Monolateral	*FAM161A*	NM_001201543.2	c.1321dup	p.(His441Profs*15)	c.1321dup	p.(His441Profs*15)	AR
P24	Male	Bilateral	*KIAA1549*	NM_020910	c.138_158del	p.(Pro47_Leu53del)	c.138_158del	p.(Pro47_Leu53del)	AR
P25	Female	Bilateral	*KLHL7*	NM_001031710.3	c.458C>T	p.(Ala153Val)	n/a	n/a	AD
P26	Male	Monolateral	*KLHL7*	NM_001031710.3	c.458C>T	p.(Ala153Val)	n/a	n/a	AD
P27	Male	Bilateral	*MAK*	NM_001242957.3	c.947dup	p.(Leu316Phefs*3)	c.814C>T	p.(Arg272*)	AR
P28	Female	Bilateral	*NRL*	NM_006177.5	c.152C>T	p.(Pro51Leu)	n/a	n/a	AD
P29	Female	Bilateral	*NRL*	NM_006177.5	c.152C>T	p.(Pro51Leu)	n/a	n/a	AD
P30	Female	Monolateral	*NRL*	NM_006177.5	c.152C>T	p.(Pro51Leu)	n/a	n/a	AD
P31	Female	Bilateral	*PDE6A*	NM_000440.3	c.1363A>T	p.(Lys455*)	c.1363A>T	p.(Lys455*)	AR
P32	Female	Monolateral	*PDE6A*	NM_000440.3	c.998+1G>A	p.(?)	c.998+1G>A	p.(?)	AR
P33	Male	Bilateral	*PDE6B*	NM_000283.4	c.[694G>A;1860C>A]	p.[(Glu232Lys);(His620Gln)]	c.1727G>A	p.(Gly576Asp)	AR
P34	Male	Monolateral	*PDE6B*	NM_000283.4	c.1107+3A>G	p.(?)	c.1107+3A>G	p.(?)	AR
P35	Male	Bilateral	*PDE6B*	NM_000283.4	c.1726G>A	p.(Gly576Ser)	c.1726G>A	p.(Gly576Ser)	AR
P36	Male	Monolateral	*PDE6B*	NM_000283.4	c.1726G>A	p.(Gly576Ser)	c.1726G>A	p.(Gly576Ser)	AR
P37	Male	Monolateral	*PDE6B*	NM_000283.4	c.1742A>G	p.(Tyr581Cys)	c.2152G>A	p.(Asp718Asn)	AR
P38	Male	Bilateral	*PDE6B*	NM_000283.4	c.1798G>A	p.(Asp600Asn)	c.1798G>A	p.(Asp600Asn)	AR
P39	Female	Bilateral	*PDE6B*	NM_000283.4	c.1798G>A	p.(Asp600Asn)	c.1798G>A	p.(Asp600Asn)	AR
P40	Male	Monolateral	*PDE6B*	NM_000283.4	c.1798G>A	p.(Asp600Asn)	c.1798G>A	p.(Asp600Asn)	AR
P41	Male	Bilateral	*PDE6B*	NM_000283.4	c.385G>A	p.(Glu129Lys)	c.811G>A	p.(Glu271Lys)	AR
P42	Female	Bilateral	*PDE6B*	NM_000283.4	c.385G>A	p.(Glu129Lys)	c.811G>A	p.(Glu271Lys)	AR
P43	Female	Bilateral	*PDE6B*	NM_000283.4	c.739T>A	p.(Phe247Ile)	c.1926del	p.(Asn643Thrfs*6)	AR
P44	Male	Bilateral	*PDE6B*	NM_000283.4	c.752C>T	p.(Thr251Met)	c.2352+2C>T	p.(?)	AR
P45	Female	Monolateral	*PDE6B*	NM_000283.4	c.811G>A	p.(Glu271Lys)	c.1727G>A	p.(Gly576Asp)	AR
P46	Female	Bilateral	*PRPF3*	NM_004698.4	c.1481C>T	p.(Thr494Met)	n/a	n/a	AD
P47	Female	Bilateral	*PRPF3*	NM_004698.4	c.1481C>T	p.(Thr494Met)	n/a	n/a	AD
P48	Male	Bilateral	*PRPF3*	NM_004698.4	c.1481C>T	p.(Thr494Met)	n/a	n/a	AD
P49	Female	Bilateral	*PRPF3*	NM_004698.4	c.1481C>T	p.(Thr494Met)	n/a	n/a	AD
P50	Male	Bilateral	*PRPF3*	NM_004698.4	c.1481C>T	p.(Thr494Met)	n/a	n/a	AD
P51	Male	Bilateral	*PRPF3*	NM_004698.4	c.1481C>T	p.(Thr494Met)	n/a	n/a	AD
P52	Female	Bilateral	*PRPF3*	NM_004698.4	c.1481C>T	p.(Thr494Met)	n/a	n/a	AD
P53	Male	Bilateral	*PRPF3*	NM_004698.4	c.1481C>T	p.(Thr494Met)	n/a	n/a	AD
P54	Female	Bilateral	*PRPF3*	NM_004698.4	c.1481C>T	p.(Thr494Met)	n/a	n/a	AD
P55	Male	Monolateral	*PRPF31*	NM_015629.4	c.1145A>G	p.(Glu382Gly)	n/a	n/a	AD
P56	Female	Monolateral	*PRPF31*	NM_015629.4	c.1165C>T	p.(Gln389*)	n/a	n/a	AD
P57	Male	Monolateral	*PRPF31*	NM_015629.4	c.138_139del	p.(Val48Glnfs*9)	n/a	n/a	AD
P58	Female	Monolateral	*PRPF31*	NM_015629.4	c.322+5G>A	p.(?)	n/a	n/a	AD
P59	Male	Bilateral	*PRPF31*	NM_015629.4	c.549del	p.(Glu183Aspfs*15)	n/a	n/a	AD
P60	Male	Bilateral	*PRPF31*	NM_015629.4	c.690del	p.(Ile231Serfs*8)	n/a	n/a	AD
P61	Male	Monolateral	*PRPF31*	NM_015629.4	c.690del	p.(Ile231Serfs*8)	n/a	n/a	AD
P62	Female	Bilateral	*PRPF31*	NM_015629.4	c.855+3G>C	p.(?)	n/a	n/a	AD
P63	Female	Bilateral	*PRPF31*	NM_015629.4	c.900del	p.(Leu301Trpfs*20)	n/a	n/a	AD
P64	Female	Bilateral	*PRPF31*	NM_015629.4	c.992G>A	p.(Trp331*)	n/a	n/a	AD
P65	Male	Bilateral	*PRPF31*	NM_015629.4	deletion exons 2-14	?	n/a	n/a	AD
P66	Male	Monolateral	*PRPF31*	NM_015629.4	deletion exons 2-14	?	n/a	n/a	AD
P67	Female	Bilateral	*PRPF31*	NM_015629.4	deletion exons 2-14	?	n/a	n/a	AD
P68	Male	Bilateral	*PRPF8*	NM_006445.4	c.6901C>T	p.(Pro2301Ser)	n/a	n/a	AD
P69	Female	Bilateral	*PRPF8*	NM_006445.4	c.6901C>T	p.(Pro2301Ser)	n/a	n/a	AD
P70	Female	Bilateral	*PRPF8*	NM_006445.4	c.6901C>T	p.(Pro2301Ser)	n/a	n/a	AD
P71	Female	Bilateral	*PRPF8*	NM_006445.4	c.6926A>G	p.(His2309Arg)	n/a	n/a	AD
P72	Female	Bilateral	*PRPF8*	NM_006445.4	c.6938A>C	p.(His2313Pro)	n/a	n/a	AD
P73	Female	Bilateral	*PRPF8*	NM_006445.4	c.6938A>C	p.(His2313Pro)	n/a	n/a	AD
P74	Female	Bilateral	*PRPF8*	NM_006445.4	c.6938A>C	p.(His2313Pro)	n/a	n/a	AD
P75	Female	Monolateral	*PRPF8*	NM_006445.4	c.6938A>C	p.(His2313Pro)	n/a	n/a	AD
P76	Male	Monolateral	*PRPH2*	NM_000322.5	c.458A>G	p.(Lys153Arg)	n/a	n/a	AD
P77	Male	Monolateral	*PRPH2*	NM_000322.5	c.535T>G	p.(Trp179Gly)	n/a	n/a	AD
P78	Male	Monolateral	*PRPH2*	NM_000322.5	c.668T>A	p.(Ile223Asn)	n/a	n/a	AD
P79	Female	Monolateral	*RAX2*	NM_032753	c.-68_216del	?	c.-68_216del	?	AR
P80	Male	Bilateral	*RBP3*	NM_002900.3	c.3238G>A	p.(Asp1080Asn)	c.3238G>A	p.(Asp1080Asn)	AR
P81	Female	Bilateral	*REEP6*	NM_001329556.3	c.210-2A>G	p.(?)	c.210-2A>G	p.(?)	AR
P82	Female	Monolateral	*RHO*	NM_000539.3	c.1028G>A	p.(Ser343Asn)	n/a	n/a	AD
P83	Female	Bilateral	*RHO*	NM_000539.3	c.1040C>T	p.(Pro347Leu)	n/a	n/a	AD
P84	Female	Bilateral	*RHO*	NM_000539.3	c.1040C>T	p.(Pro347Leu)	n/a	n/a	AD
P85	Male	Bilateral	*RHO*	NM_000539.3	c.1040C>T	p.(Pro347Leu)	n/a	n/a	AD
P86	Male	Bilateral	*RHO*	NM_000539.3	c.1040C>T	p.(Pro347Leu)	n/a	n/a	AD
P87	Male	Bilateral	*RHO*	NM_000539.3	c.1040C>T	p.(Pro347Leu)	n/a	n/a	AD
P88	Female	Monolateral	*RHO*	NM_000539.3	c.1040C>T	p.(Pro347Leu)	n/a	n/a	AD
P89	Female	Bilateral	*RHO*	NM_000539.3	c.1045T>C	p.(*349Glnext*51)	n/a	n/a	AD
P90	Female	Bilateral	*RHO*	NM_000539.3	c.181G>A	p.(Val61Ile)	n/a	n/a	AD

P91	Female	Bilateral	*RHO*	NM_000539.3	c.190C>T	p.(Gln64*)	n/a	n/a	AD
P92	Female	Bilateral	*RHO*	NM_000539.3	c.364G>A	p.(Glu122Lys)	n/a	n/a	AD
P93	Female	Bilateral	*RHO*	NM_000539.3	c.403C>T	p.(Arg135Trp)	n/a	n/a	AD
P94	Female	Bilateral	*RHO*	NM_000539.3	c.403C>T	p.(Arg135Trp)	n/a	n/a	AD
P95	Female	Bilateral	*RHO*	NM_000539.3	c.403C>T	p.(Arg135Trp)	n/a	n/a	AD
P96	Female	Monolateral	*RHO*	NM_000539.3	c.403C>T	p.(Arg135Trp)	n/a	n/a	AD
P97	Male	Bilateral	*RHO*	NM_000539.3	c.403C>T	p.(Arg135Trp)	n/a	n/a	AD
P98	Female	Bilateral	*RHO*	NM_000539.3	c.403C>T	p.(Arg135Trp)	n/a	n/a	AD
P99	Female	Bilateral	*RHO*	NM_000539.3	c.403C>T	p.(Arg135Trp)	n/a	n/a	AD
P100	Female	Bilateral	*RHO*	NM_000539.3	c.403C>T	p.(Arg135Trp)	n/a	n/a	AD
P101	Female	Bilateral	*RHO*	NM_000539.3	c.473C>A	p.(Ala158Asp)	n/a	n/a	AD
P102	Female	Monolateral	*RHO*	NM_000539.3	c.473C>A	p.(Ala158Asp)	n/a	n/a	AD
P103	Female	Bilateral	*RHO*	NM_000539.3	c.473C>A	p.(Ala158Asp)	n/a	n/a	AD
P104	Female	Bilateral	*RHO*	NM_000539.3	c.473C>A	p.(Ala158Asp)	n/a	n/a	AD
P105	Female	Bilateral	*RHO*	NM_000539.3	c.473C>A	p.(Ala158Asp)	n/a	n/a	AD
P106	Male	Bilateral	*RHO*	NM_000539.3	c.473C>A	p.(Ala158Asp)	n/a	n/a	AD
P107	Male	Bilateral	*RHO*	NM_000539.3	c.473C>A	p.(Ala158Asp)	n/a	n/a	AD
P108	Female	Bilateral	*RHO*	NM_000539.3	c.473C>A	p.(Ala158Asp)	n/a	n/a	AD
P109	Female	Bilateral	*RHO*	NM_000539.3	c.491C>T	p.(Ala164Val)	n/a	n/a	AD
P110	Female	Monolateral	*RHO*	NM_000539.3	c.509C>G	p.(Pro170Arg)	n/a	n/a	AD
P111	Female	Monolateral	*RHO*	NM_000539.3	c.509C>G	p.(Pro170Arg)	n/a	n/a	AD
P112	Female	Bilateral	*RHO*	NM_000539.3	c.50C>G	p.(Thr17Arg)	n/a	n/a	AD
P113	Male	Bilateral	*RHO*	NM_000539.3	c.541G>A	p.(Glu181Lys)	n/a	n/a	AD
P114	Female	Monolateral	*RHO*	NM_000539.3	c.560G>T	p.(Cys187Phe)	n/a	n/a	AD
P115	Female	Bilateral	*RHO*	NM_000539.3	c.560G>T	p.(Cys187Phe)	n/a	n/a	AD
P116	Male	Monolateral	*RHO*	NM_000539.3	c.568G>T	p.(Asp190Tyr)	n/a	n/a	AD
P117	Female	Bilateral	*RHO*	NM_000539.3	c.644C>T	p.(Pro215Leu)	n/a	n/a	AD
P118	Female	Bilateral	*RHO*	NM_000539.3	c.644C>T	p.(Pro215Leu)	n/a	n/a	AD
P119	Male	Bilateral	*RHO*	NM_000539.3	c.644C>T	p.(Pro215Leu)	n/a	n/a	AD
P120	Male	Bilateral	*RHO*	NM_000539.3	c.644C>T	p.(Pro215Leu)	n/a	n/a	AD
P121	Male	Bilateral	*RP1*	NM_006269.2	c.2029C>T	p.(Arg677*)	n/a	n/a	AD
P122	Female	Bilateral	*RP1*	NM_006269.2	c.2029C>T	p.(Arg677*)	n/a	n/a	AD
P123	Male	Monolateral	*RP1*	NM_006269.2	c.2029C>T	p.(Arg677*)	n/a	n/a	AD
P124	Female	Bilateral	*RP1*	NM_006269.2	c.2219C>G	p.(Ser740*)	n/a	n/a	AD
P125	Female	Bilateral	*RP1*	NM_006269.2	c.2219C>G	p.(Ser740*)	n/a	n/a	AD
P126	Female	Monolateral	*RP1*	NM_006269.2	c.2219C>G	p.(Ser740*)	n/a	n/a	AD
P127	Female	Monolateral	*RP1*	NM_006269.2	c.2219C>G	p.(Ser740*)	n/a	n/a	AD
P128	Male	Bilateral	*RP1*	NM_006269.2	c.2447dup	p.(Asn816Lysfs*9)	n/a	n/a	AD
P129	Female	Bilateral	*RP1*	NM_006269.2	c.2978del	p.(Ser993Phefs*20)	n/a	n/a	AD
P130	Male	Bilateral	*RP2*	NM_006915.3	c.535C>T	p.(Pro179Ser)	n/a	n/a	XLR
P131	Male	Bilateral	*RPGR*	NM_001034853.2	c.154G>A	p.(Gly52Arg)	n/a	n/a	XLR
P132	Male	Monolateral	*RPGR*	NM_001034853.2	c.155-2A>G	p.(?)	n/a	n/a	XLR
P133	Male	Monolateral	*RPGR*	NM_001034853.2	c.2322del	p.(Arg775Glyfs*40)	n/a	n/a	XLR
P134	Male	Bilateral	*RPGR*	NM_001034853.2	c.2412_2418del	p.(Gly805Lysfs*8)	n/a	n/a	XLR
P135	Male	Bilateral	*RPGR*	NM_001034853.2	c.2570_2571del	p.(Lys857Argfs*221)	n/a	n/a	XLR
P136	Male	Monolateral	*RPGR*	NM_001034853.2	c.2760_2761del	p.(Glu922Glyfs*156)	n/a	n/a	XLR
P137	Male	Monolateral	*RPGR*	NM_001034853.2	c.2760_2761del	p.(Glu922Glyfs*156)	n/a	n/a	XLR
P138	Male	Monolateral	*RPGR*	NM_001034853.2	c.748T>C	p.(Cys250Arg)	n/a	n/a	XLR
P139	Male	Bilateral	*SNRNP200*	NM_014014.5	c.2041C>T	p.(Arg681Cys)	n/a	n/a	AD
P140	Male	Bilateral	*SNRNP200*	NM_014014.5	c.2041C>T	p.(Arg681Cys)	n/a	n/a	AD
P141	Male	Bilateral	*SNRNP200*	NM_014014.5	c.2041C>T	p.(Arg681Cys)	n/a	n/a	AD
P142	Male	Bilateral	*SNRNP200*	NM_014014.5	c.2580G>C	p.(Gln860His)	n/a	n/a	AD
P143	Female	Bilateral	*SNRNP200*	NM_014014.5	c.3191A>G	p.(Gln1064Arg)	c.3191A>G	p.(Gln1064Arg)	AR
P144	Female	Monolateral	*SNRNP200*	NM_014014.5	c.3191A>G	p.(Gln1064Arg)	c.3191A>G	p.(Gln1064Arg)	AR
P145	Male	Monolateral	*USH2A*	NM_206933.4	c.1040A>G	p.(Asp347Gly)	c.9949C>T	p.(Arg3317Cys)	AR
P146	Female	Bilateral	*USH2A*	NM_206933.4	c.10712C>T	p.(Thr3571Met)	c.14134-5T>C	p.(?)	AR
P147	Female	Bilateral	*USH2A*	NM_206933.4	c.10712C>T	p.(Thr3571Met)	c.14219C>A	p.(Ala4740Asp)	AR
P148	Male	Bilateral	*USH2A*	NM_206933.4	c.10712C>T	p.(Thr3571Met)	c.14219C>A	p.(Ala4740Asp)	AR
P149	Female	Bilateral	*USH2A*	NM_206933.4	c.10712C>T	p.(Thr3571Met)	c.4711G>C	p.(Ala1571Pro)	AR
P150	Female	Bilateral	*USH2A*	NM_206933.4	c.10817T>C	p.(Leu3606Pro)	c.10699del	p.(Leu3567*)	AR
P151	Male	Bilateral	*USH2A*	NM_206933.4	c.10817T>C	p.(Leu3606Pro)	c.9676C>T	p.(Arg3226*)	AR
P152	Male	Bilateral	*USH2A*	NM_206933.4	c.11156G>A	p.(Arg3719His)	c.4758+3A>G	p.(?)	AR
P153	Male	Bilateral	*USH2A*	NM_206933.4	c.11713C>T	p.(Arg3905Cys)	c.13385T>C	p.(Ile4462Thr)	AR
P154	Male	Bilateral	*USH2A*	NM_206933.4	c.11713C>T	p.(Arg3905Cys)	c.949C>A	p.(Arg317=)	AR
P155	Male	Bilateral	*USH2A*	NM_206933.4	c.12067-2A>G	p.(?)	c.3062G>T	p.(Cys1021Phe)	AR
P156	Male	Bilateral	*USH2A*	NM_206933.4	c.12574C>T	p.(Arg4192Cys)	c.12574C>T	p.(Arg4192Cys)	AR
P157	Female	Bilateral	*USH2A*	NM_206933.4	c.13022G>A	p.(Cys4341Tyr)	c.232T>G	p.(Phe78Val)	AR
P158	Male	Bilateral	*USH2A*	NM_206933.4	c.13385T>C	p.(Ile4462Thr)	c.13385T>C	p.(Ile4462Thr)	AR
P159	Female	Monolateral	*USH2A*	NM_206933.4	c.14219C>A	p.(Ala4740Asp)	c.10322A>G	p.(Glu3441Gly)	AR
P160	Female	Monolateral	*USH2A*	NM_206933.4	c.14219C>A	p.(Ala4740Asp)	c.12067-2A>G	p.(?)	AR
P161	Male	Bilateral	*USH2A*	NM_206933.4	c.14286C>A	p.(Asn4762Lys)	c.7524del	p.(Arg2509Glyfs*19)	AR
P162	Male	Bilateral	*USH2A*	NM_206933.4	c.15125G>A	p.(Trp5042*)	c.1478A>G	p.(Tyr493Cys)	AR
P163	Male	Bilateral	*USH2A*	NM_206933.4	c.15322del	p.(Arg5108Glyfs*6)	c.12575G>A	p.(Arg4192His)	AR
P164	Female	Bilateral	*USH2A*	NM_206933.4	c.1547G>T	p.(Gly516Val)	c.232T>G	p.(Phe78Val)	AR
P165	Male	Monolateral	*USH2A*	NM_206933.4	c.2276G>T	p.(Cys759Phe)	c.10429T>C	p.(Ser3477Pro)	AR
P166	Female	Bilateral	*USH2A*	NM_206933.4	c.2276G>T	p.(Cys759Phe)	c.2299del	p.(Glu767Serfs*21)	AR
P167	Female	Bilateral	*USH2A*	NM_206933.4	c.2276G>T	p.(Cys759Phe)	c.3920C>G	p.(Ser1307*)	AR
P168	Female	Monolateral	*USH2A*	NM_206933.4	c.2276G>T	p.(Cys759Phe)	c.4717C>T	p.(Gln1573*)	AR
P169	Female	Bilateral	*USH2A*	NM_206933.4	c.2276G>T	p.(Cys759Phe)	c.907C>T	p.(Arg303Cys)	AR
P170	Female	Monolateral	*USH2A*	NM_206933.4	c.2299del	p.(Glu767Serfs*21)	c.12371C>T	p.(Pro4124Leu)	AR
P171	Female	Monolateral	*USH2A*	NM_206933.4	c.3045C>G	p.(His1015Gln)	c.6992G>A	p.(Gly2331Glu)	AR
P172	Male	Bilateral	*USH2A*	NM_206933.4	c.3920C>G	p.(Ser1307*)	c.8254G>A	p.(Gly2752Arg)	AR
P173	Male	Monolateral	*USH2A*	NM_206933.4	c.3920C>G	p.(Ser1307*)	c.8254G>A	p.(Gly2752Arg)	AR
P174	Female	Bilateral	*USH2A*	NM_206933.4	c.4106C>T	p.(Ser1369Leu)	c.14174G>A	p.(Trp4725*)	AR
P175	Male	Bilateral	*USH2A*	NM_206933.4	c.4988-2A>G	p.(?)	c.6937G>T	p.(Gly2313Cys)	AR
P176	Male	Monolateral	*USH2A*	NM_206933.4	c.5776+1G>A	p.(?)	c.3920C>G	p.(Ser1307*)	AR
P177	Male	Monolateral	*USH2A*	NM_206933.4	c.6169C>T	p.(Gln2057*)	c.908G>A	p.(Arg303His)	AR
P178	Male	Bilateral	*USH2A*	NM_206933.4	c.6721C>A	p.(Pro2241Thr)	c.14977_14978del	p.(Phe4993Profs*7)	AR
P179	Female	Bilateral	*USH2A*	NM_206933.4	c.908G>A	p.(Arg303His)	c.13655G>A	p.(Trp4552*)	AR
